# Effects of Combined Surface and In-Depth Absorption on Ignition of PMMA

**DOI:** 10.3390/ma9100820

**Published:** 2016-10-05

**Authors:** Junhui Gong, Yixuan Chen, Jing Li, Juncheng Jiang, Zhirong Wang, Jinghong Wang

**Affiliations:** 1 College of Safety Science and Engineering, Nanjing Tech University, Nanjing 210009, China; gjh9896@njtech.edu.cn (J.G.); chenxi1991@njtech.edu.cn (Y.C.); jcjiang@njtech.edu.cn (J.J.); arain@njtech.edu.cn (J.W.); 2 Department of Fire Science & Professional Studies, University of New Haven, West Haven, CT 06516, USA; jli@newhaven.edu

**Keywords:** surface absorption, in-depth absorption, ignition, thermal degradation, PMMA

## Abstract

A one-dimensional numerical model and theoretical analysis involving both surface and in-depth radiative heat flux absorption are utilized to investigate the influence of their combination on ignition of PMMA (Polymethyl Methacrylate). Ignition time, transient temperature in a solid and optimized combination of these two absorption modes of black and clear PMMA are examined to understand the ignition mechanism. Based on the comparison, it is found that the selection of constant or variable thermal parameters of PMMA barely affects the ignition time of simulation results. The linearity between $t_{ig}^{-0.5}$ and heat flux does not exist anymore for high heat flux. Both analytical and numerical models underestimate the surface temperature and overestimate the temperature in a solid beneath the heat penetration layer for pure in-depth absorption. Unlike surface absorption circumstances, the peak value of temperature is in the vicinity of the surface but not on the surface for in-depth absorption. The numerical model predicts the ignition time better than the analytical model due to the more reasonable ignition criterion selected. The surface temperature increases with increasing incident heat flux. Furthermore, it also increases with the fraction of surface absorption and the radiative extinction coefficient for fixed heat flux. Finally, the combination is optimized by ignition time, temperature distribution in a solid and mass loss rate.

## 1. Introduction

Ignition of a solid combustible followed by thermal degradation under external incident heat flux determines the subsequent fire propagation, and thus has been investigated extensively in the last few decades because of its great importance. When heated, the combustible undergoes pyrolysis, involving thermodynamics [1,2,3,4,5,6,7], chemical kinetics [1,2,3,4,5,6,7], transmission of yield volatiles [8,9,10], volume deformation [8,11], generation of char [3,5,7,12] and phase transformation [11], etc. Thermal decomposition in a solid has been studied by numerous pyrolysis models, analytical or numerical, and the combustion process in gas after ignition has also been relatively well studied by solving a series of governing equations coupled with appropriate boundary and initial conditions [13,14]. However, the ignition mechanism connecting solid pyrolysis and gas phase combustion is still not adequately understood due to its inherent complexity.

Ignition, spontaneous or piloted, occurs only when some sorts of ignition criteria are satisfied. Four criteria can be found in the literature and were summarized by Vermesi [15], namely critical temperature, critical mass flux, critical energy and time-energy squared. The critical temperature is reliable for a thermally thick solid exposed to a constant irradiation (e.g., Ref. [16]). It is assumed that ignition occurs when a critical surface temperature is achieved, which is defined as a characteristic parameter of specified material, indicating that it is an invariant constant. The dependence of thermal parameters on temperature and chemical reaction in a solid are neglected. The total incident heat flux is absorbed at the surface, which means the solid is opaque for radiation and the surface absorptivity coefficient is equal to 1. This criterion is extensively used in analytical model for simplification. The main criticism of this criterion includes an ambiguous ignition mechanism, neglect of in-depth absorption of irradiation and the fact that the surface temperature of a solid increases with increasing incident heat flux, which is validated by experimental measurement (e.g., [17,18]). Considering the lower flammability limit of pyrolyzate, critical mass loss rate is proposed as a more reasonable ignition criterion. It takes into account the thermal degradation in a solid and is commonly employed in numerical models. With a constant mass flux criterion, the surface temperature at ignition time increases with increasing heat flux. The interpretation is that the pyrolysis reaction is increasingly confined to a thin layer near the surface and this layer must be raised to a higher temperature to achieve the critical mass flux than at a lower radiant flux (e.g., [18]). Furthermore, Bal’s [19] work suggested the maximum temperature exists below the surface when taking in-depth absorption and surface heat loss into consideration. The depth of the peak value of temperature can be estimated by the thermal depth of radiation or conduction, depending on the combination of surface and in-depth absorption mode. Apparently, critical temperature should not be adopted especially for a translucent solid. The energy criterion provides ranges of critical energy rather than a single value for each material, which states that a sample will ignite after absorbing a certain amount of energy (e.g., [20]). Reszka [21] proposed a time-energy squared criterion when studying the ignition delay time of material under time-dependent heat flux. The ignition delay time is expressed as a function of the total energy delivered to the surface.

Almost all of these criteria adopt the surface absorption hypothesis, coupling the external incident heat flux in surface boundary conditions. This assumption is credible and reasonable for infrared opaque materials. However, for a translucent solid, like some polymers, in-depth absorption exerts its influence on thermal degradation and subsequently affects the ignition mechanism. Recently, this subject has been paid great attention in some pyrolysis models (e.g., [8,9,22]). In these models, incident heat flux is absorbed inside the material gradually and no energy is absorbed on the surface. Both surface and in-depth absorption are idealized cases, and the failure of the classical ignition theory is confirmed by experiential observation, especially under high heat flux (e.g., [23,24,25]). Jiang [26] measured the in-depth absorption coefficient of black PMMA with the water-cooled method. Surface and in-depth absorption are separately studied by an established theoretical model, and the divergence between theoretical model and experimental data demonstrates that both absorption modes are the controlling mechanism of ignition. Moreover, it was also found that in-depth absorption exists even for the presence of coated carbon black on the surface. Bal [19] and Staggs [27] numerically investigated the ignition behaviors of PMMA considering both surface and in-depth absorption, and an optimized combination was suggested. Also, it was found that the traditional coating of black carbon added on the sample does not cancel in-depth radiation absorption but rather its effect is to absorb around 35% of the incoming radiation at the surface. Delichatsios [28] modified the classical ignition theory by taking both absorption modes into account and provided some useful results. Although these studies provided exploratory investigation on effects of combination of surface and in-depth absorption on pyrolysis or ignition, numerous important issues remain unsolved, like methods on determination of optimized combination, influence of the char layer generated during thermal degradation, effects of spectral distribution of radiation on the radiative properties due to the non-grey spectral nature of polymers, variation of combination during pyrolysis caused by changing optical characteristics of surface etc.

In this work, both surface and in-depth absorption of incident heat flux are considered using analytical and numerical methods to examine the ignition mechanism of PMMA. Optimized combinations obtained with the theoretical and numerical models are discussed and compared. The reasonability of utilization of constant thermal parameters in a numerical model, critical ignition temperature, critical mass flux, and a constant combination during the entire pyrolysis process are also explored. Both black and clear PMMA experimental measurements are employed to validate the theoretical and numerical model. Additionally, the effect of a coated black carbon surface layer on combination and ignition is also studied.

## 2. Theoretical Analysis

Considering the heat transfer, including both thermal conduction and in-depth radiation, in one dimensional translucent polymer, the energy balance equation can be expressed as:

(1) $$\rho_{s}C_{s}\frac{\partial T_{s}}{\partial t}=\frac{\partial }{\partial x}\left(k_{s}\frac{\partial T_{s}}{\partial x}\right)+\left(1-\lambda \right){\dot{q}}_{ext}^{\prime \prime }\kappa e^{-\kappa x}$$

Initial and boundary conditions:

(2) $$\left\{ \begin{array}{l} T_{s}\left(x,0\right)=T_{0}\\ {-k_{s}\frac{\partial T_{s}}{\partial x}|}_{x=0}=\lambda {\dot{q}}_{ext}^{\prime \prime }-\varepsilon \sigma \left(T_{s}^{4}-T_{0}^{4}\right)-h_{C}\left(T_{s}-T_{0}\right)\\ T_{s}\left(\infty ,t\right)=T_{0} \end{array}\right.$$

where $\rho_{s}$ is density of solid, $C_{s}$ is specific heat, $T_{s}$ is temperature, t is time, $k_{s}$ is thermal conductivity, x is spatial variable in thickness direction, ${\dot{q}}_{ext}^{\prime \prime }$ is the applied heat flux, λ is the fraction of imposed radiant heat flux absorbed at the surface, κ is the in-depth absorption coefficient (or radiative extinction coefficient), ε is emissivity of surface, σ is the Stefan-Boltzmann constant, $h_{C}$ is the surface convection coefficient, $T_{0}$ is initial and ambient temperature. In the analytical model, decomposition reactions, dependences of thermal parameters on temperature and mass transfer of volatiles inside the PMMA are neglected for simplification based on the fact that these processes have negligible influences on ignition.

With a defined relative temperature,

(3) $$\theta =T_{s}-T_{0}$$

(4) $$\theta =\left(1-\lambda \right)\theta_{1}+\lambda \theta_{2}$$

the problem can be decomposed as a superposition of two simpler problems, which are equivalent to two idealized cases (e.g., [28]):

1. In-depth heating without surface heat loss

(5) $$\frac{\partial \theta_{1}}{\partial t}=\alpha \frac{\partial^{2}\theta_{1}}{\partial x^{2}}+{\dot{q}}_{ext}^{\prime \prime }\kappa e^{-\kappa x}$$

Initial and boundary conditions:

(6) $$\left\{ \begin{array}{l} \theta_{1}\left(x,0\right)=0\\ {-k_{s}\frac{\partial \theta_{1}}{\partial x}|}_{x=0}=0\\ \theta_{1}\left(\infty ,t\right)=0 \end{array}\right.$$

Similar work has been performed in Ref. [26], and an analytical solution is obtained by Laplace transforms:

(7) $$\theta_{1}\left(x,t\right)=\frac{{\dot{q}}_{ext}^{\prime \prime }}{2\kappa k_{s}}\left\{4\kappa \sqrt{\alpha t}ierfc\left(\frac{x}{2\sqrt{\alpha t}}\right)+e^{\kappa^{2}\alpha t+\kappa x}erfc\left(\kappa \sqrt{\alpha t}+\frac{x}{2\sqrt{\alpha t}}\right)+e^{\kappa^{2}\alpha t-\kappa x}erfc\left(\kappa \sqrt{\alpha t}-\frac{x}{2\sqrt{\alpha t}}\right)-2e^{-\kappa x}\right\}$$

2. Surface heating with surface heat loss

(8) $$\frac{\partial \theta_{2}}{\partial t}=\alpha \frac{\partial^{2}\theta_{2}}{\partial x^{2}}$$

Initial and boundary conditions:

(9) $$\left\{ \begin{array}{l} \theta_{2}\left(x,0\right)=0\\ {-\frac{k_{s}}{\lambda }\frac{\partial \theta_{2}}{\partial x}|}_{x=0}={\dot{q}}_{ext}^{\prime \prime }-\frac{h_{C}+\varepsilon h_{R}}{\lambda }\theta_{2}\\ \theta_{2}\left(\infty ,t\right)=0 \end{array}\right.$$

where $h_{R}$ is the surface radiation approximation coefficient by introducing a hypothesis $\sigma \left(T_{s}^{4}-T_{0}^{4}\right)=h_{R}\left(T_{s}-T_{0}\right)$ [26]. Also, the solution can be obtained as:

(10) $$\theta_{2}\left(x,t\right)=\frac{\lambda {\dot{q}}_{ext}^{\prime \prime }}{Hk_{s}}\left[erfc\left(\frac{x}{2\sqrt{\alpha t}}\right)-e^{H^{2}\alpha t+Hx}erfc\left(\frac{x}{2\sqrt{\alpha t}}+H\sqrt{\alpha t}\right)\right]$$

where $H=\left(h_{C}+\varepsilon h_{R}\right)/k_{s}$, $h_{C}=10W/m^{2}K,\;h_{R}=20W/m^{2}K$ [26].

Combining Equations (3), (4), (7) and (10), transient temperature in condensed solid can be derived. Furthermore, surface temperature can also be obtained when x=0:

(11) $$\theta \left(0,t\right)=\frac{\left(1-\lambda \right){\dot{q}}_{ext}^{\prime \prime }}{\kappa k_{s}}\left[2\kappa \sqrt{\frac{\alpha t}{\pi }}+e^{\kappa^{2}\alpha t}erfc\left(\kappa \sqrt{\alpha t}\right)-1\right]+\frac{\lambda {\dot{q}}_{ext}^{\prime \prime }}{Hk_{s}}\left[1-e^{H^{2}\alpha t}erfc\left(H\sqrt{\alpha t}\right)\right]$$

In classical ignition theoretical model, critical ignition temperature is used, $T_{ig}$. Heat loss at the surface is ignored and the heat flux is only absorbed by surface absorption, namely λ=1, $h_{C}=h_{R}=0$. With these assumptions, the exact solution of Equations (8) and (9) is:

(12) $$\theta_{2}\left(x,t\right)=\frac{{\dot{q}}_{ext}^{\prime \prime }}{\sqrt{k\rho c}}\int_{0}^{t}erfc\left(\frac{x}{2\sqrt{\alpha t}}\right)\cdot {\left(t-\tau \right)}^{-0.5}d\tau$$

When ignition occurs, $x=0,T_{s}=T_{ig}$, the classical correlation is obtained:

(13) $$\frac{1}{\sqrt{t_{ig,\lambda =1}}}=\frac{2}{\sqrt{\pi }\cdot \sqrt{k_{s}\rho c}}\frac{{\dot{q}}_{ext}^{\prime \prime }}{\left(T_{ig}-T_{0}\right)}$$

Some other researchers modified this expression by accounting for the critical heat flux (e.g., [28]):

(14) $$\frac{1}{\sqrt{t_{ig,\lambda =1}}}=\frac{2}{\sqrt{\pi }\cdot \sqrt{k_{s}\rho c}}\frac{\left({\dot{q}}_{ext}^{\prime \prime }-0.64{\dot{q}}_{cr}^{\prime \prime }\right)}{\left(T_{ig}-T_{0}\right)}$$

While for in-depth absorption scenario, λ=0, Delichatsios [28] simplified $\theta_{1}$ for low and high heat fluxes, lower and higher than 80 kW/m^2^, respectively.

For low heat fluxes, the correlation can be derived from Equation (14c) of Ref. [28]:

(15) $$\frac{1}{\sqrt{t_{ig,\lambda =0}}}=\frac{2\kappa \sqrt{\alpha }}{\sqrt{\pi }+2\kappa \sqrt{\alpha }/t_{ig,\lambda =1}^{-0.5}}$$

For high heat fluxes, the correlation can be derived from Equation (14d) of Ref. [28]:

(16) $$\frac{1}{\sqrt{t_{ig,\lambda =0}}}=-\kappa \sqrt{\frac{\alpha }{\pi }}+\sqrt{\frac{\alpha \kappa^{2}}{\pi }+2\kappa \sqrt{\frac{\alpha }{\pi }}t_{ig,\lambda =1}^{-0.5}}\;$$

When 0<λ<1, both in-depth and surface absorption exists, and the correlation can be derived from Equation (14e) of Ref. [28] for low heat flux:

(17) $$\frac{1}{\sqrt{t_{ig,\lambda }}}=\frac{2\kappa \sqrt{\alpha }}{\sqrt{\pi }\left(1-\lambda \right)+2\kappa \sqrt{\alpha }/t_{ig,\lambda =1}^{-0.5}}$$

Additionally, Staggs [27] proposed the following formulation to qualitatively estimate the effects of in-depth absorption on ignition:

(18) $$\frac{1}{\sqrt{t_{ig,\lambda }}}\sim \lambda \frac{\sqrt{2}{\dot{q}}_{ext}^{\prime \prime }}{\sqrt{k_{s}\rho c}\left(T_{ig}-T_{\infty }\right)}$$

which indicates that $1/\sqrt{t_{ig}}$ will reduce in direct proportion to λ.

## 3. Numerical Simulation

### 3.1. Model

A numerical model considering both in-depth and surface absorption, previously developed by an author (e.g., [29]), is employed in this study to examine the ignition process of PMMA. The radiation emitted by the heater is partially reflected by the surface of PMMA, characterized as reflectivity r. A fraction of the rest energy, λ, is absorbed by the surface. The remaining fraction, (1−λ), penetrating the surface is absorbed in the interior of PMMA during the attenuation. The energy conservation equation accounting for this in-depth absorption phenomenon can be expressed as [29]:

(19) $$\rho_{s}C_{s}\frac{\partial T_{s}}{\partial t}=\frac{\partial }{\partial x}\left(k_{s}\frac{\partial T_{s}}{\partial x}\right)+\left(1-r\right)\left(1-\lambda \right){\dot{q}}_{ext}^{\prime \prime }\kappa e^{-\kappa x}+\rho_{s}S_{v}\left[\Delta H_{v}+\left(T_{s}-T_{0}\right)\left(C_{s}-C_{g}\right)\right]-{\dot{m}}^{\prime \prime }C_{g}\frac{\partial T_{s}}{\partial x}$$

where $S_{v}$ is the rate of volatiles generation in a solid, $\Delta H_{v}$ is the heat of decomposition, $C_{g}$ is specific heat of gas and ${\dot{m}}^{\prime \prime }$ is the mass flux in the controlled volume. The radiation absorbed by the surface is involved in the boundary condition (e.g., [30]):

(20) $$\left(1-r\right)\lambda {\dot{q}}_{ext}^{\prime \prime }-\varepsilon \sigma \left(T_{s}^{4}-T_{0}^{4}\right)-h_{C}\left(T_{s}-T_{0}\right)=-k_{s}\frac{\partial T_{s}}{\partial x}$$

The transient total mass loss rate can be calculated by the simulation results:

(21) $${\dot{m}}^{\prime \prime }\left(x\right)=\int_{0}^{x}-S_{v}\rho_{s}dx$$

Volume regression, variation of top surface convection coefficient, kinetics and thermodynamics of thermal degradation are all considered in the model. More detailed information, including selection of parameters, reasonableness of the averaged radiation extinction coefficient, simulation strategy, and validation of the capability of model, etc. can be found in Ref. [29].

### 3.2. Simulation Parameters

Black and clear PMMA are selected for simulation based on the fact that there is available experimental data focusing on ignition under heat flux up to 200 kW/m^2^ in literatures (e.g., [18,19,23,26]). Relevant input parameters of PMMA are tabulated in Table 1.

## 4. Results and Discussion

### 4.1. Ignition Time of PMMA

In classical ignition theory and analytical model, the material is assumed to be thermally inertial and the properties of material are invariants for simplification, like density, thermal conductivity and heat capacity etc. While in numerical model, these input parameters can be constant or temperature-dependent. The later one is used in this paper if the variable properties are accessible in the literature.

**Figure 1:**
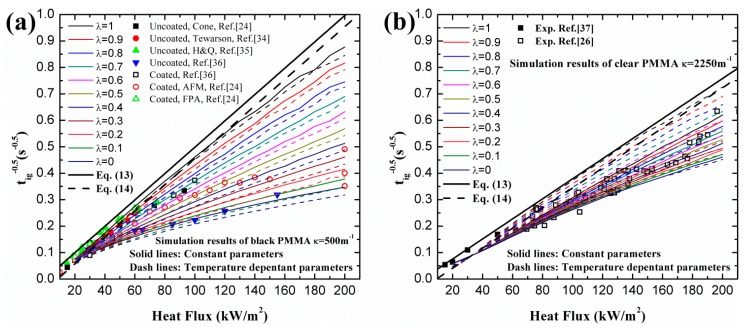
Effects of temperature-dependent parameters on ignition time of PMMA: (**a**) black; (**b**) clear.

Even though the capability of the numerical model has been validated in Ref. [29], the effects of input parameters of PMMA on ignition time of simulation results need to be investigated. Figure 1 illustrates the influence of input properties of black and clear PMMA on ignition predictions. In Figure 1a (e.g., [23,33,34,35]), with fixed λ and κ, little discrepancy exists between the simulation results of constant parameters, solid lines, and temperature-dependent ones, dash lines. This conclusion coincides with that of Bal [19]. Also, this is contrary to the suggestion in ref. [26] that the increasing thermal conductivity and the specific heat at higher temperatures would bring further curvature to $1/\sqrt{t_{ig}}$ at high heat fluxes. In Figure 1b (e.g., [25,36]), a similar phenomenon can be observed for clear PMMA. Both cases assert that the effects of temperature-dependent material properties on ignition time can be neglected. This can be inferred by the fact that the ignition in numerical model is determined by critical mass flux, not ignition temperature. ${\dot{m}}_{cri}^{\prime \prime }$, 2.42 for black PMMA and 2.5 for clear PMMA is much lower than the value of the steady stage of pyrolysis (e.g., [29]), and the variation of thermal parameters during this early stage has little effect on mass loss rate, and consequently the ignition time. After ignition, this variation in thermal parameters should be taken into consideration due to the violent degradation reaction in a solid caused by enhanced heat feedback from flame and accumulated heat from heater.

Additionally, in Figure 1, λ has significant effects on ignition time for both black and clear PMMA when it increases from 0, in-depth absorption, to 1, surface absorption. Comparison between experimental measurements and simulation results indicates that classical ignition theory cannot be applied for large heat fluxes. An optimized λ should exist in predicting the ignition time when in-depth absorption is considered, which will be presented in later section of this study. The fixed κ in Figure 1 is selected according to experiments (e.g., [6,26]).

**Figure 2:**
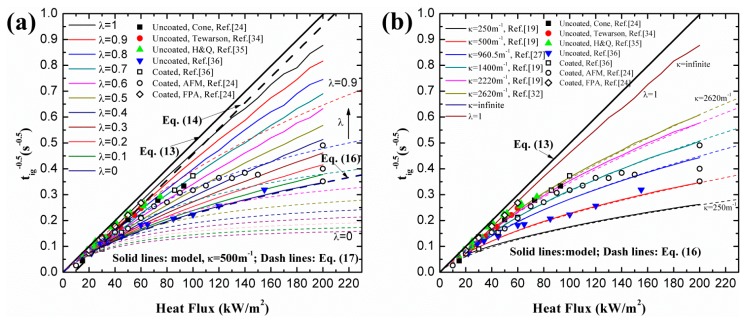
Effects of λ and κ on ignition time of black PMMA: (**a**) different λ; (**b**) different κ.

With different λ and κ, simulation results and analytical predictions of black PMMA are plotted in Figure 2. Coated surface experimental data is utilized based on the fact that some in-depth absorption occurs even in the presence of carbon black on the surface (e.g., [26]). In the analytical model, Equation (17) demonstrates the relationship between $t_{ig,\lambda }^{-0.5}$ and $t_{ig,\lambda =1}^{-0.5}$ under high heat flux. In Figure 2a, the simulation and analytical curves approach the classical ignition theory line, Equations (13) and (14), when λ increases from 0 to 1. This trend also verifies the qualitative correlation between $t_{ig,\lambda }^{-0.5}$ and λ, namely Equation (18).

**Figure 3:**
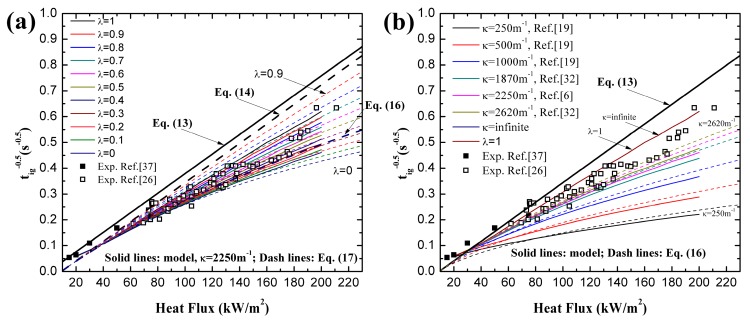
Effects of λ and κ on ignition time of clear PMMA: (**a**) different λ; (**b**) different κ.

In Figure 2b, numerical and analytical results are compared with different κ for in-depth absorption, λ = 0. Both of them approach the classical ignition theory line as κ increases from 250 m^−1^ to infinite, as described in Equation (16). This implies that extinction coefficient affects the ignition time greatly for in-depth absorption, which agrees with the conclusions of previous literature (e.g., [19,23,26,27,35]). λ = 1 and κ = ∞ get exactly the same simulation results, which indicates that when κ gets large enough the radiation penetration depth is close to 0. It should be noted that the agreement between the numerical and analytical result in Figure 2b is fairly good. However, a relative large discrepancy exists in Figure 2a. Three main reasons caused this divergence. The first is that Equation (17) is derived for relatively low heat flux, about lower than 80 kW/m^2^ (e.g., [28]), considering both in-depth and surface absorption. For larger heat flux, the accuracy of this formulation decreases. Another reason is that critical mass loss rate is used for the numerical model, which is more reasonable and reliable, while for the analytical model, a constant ignition temperature is employed. The third reason is the constant value of the surface convection coefficient, *h_C_*, and surface radiation approximation coefficient, *h_R_*, used in the analytical model (e.g., [26]). Both of them are actually temperature-dependent in numerical model. In analytical model (e.g., [29]), *h_C_* = 10 J/s·m^2^·K and *h_R_* = 20 J/s·m^2^·K provide acceptable accuracy for relatively low heat flux. However, for larger heat flux, the utilization of these constant values underestimates surface heat loss by convection and radiation due to the large surface temperature. Consequently, more heat remaining in the condensed phase leads to shorter ignition time and the discrepancy increases as the heat flux becomes larger. Equation (16) provides the necessary accuracy in predicting ignition time only for pure in-depth absorption. The same procedure was conducted for clear PMMA and similar conclusions can be obtained in Figure 3.

### 4.2. Transient Temperature Distribution in a Solid

**Figure 4:**
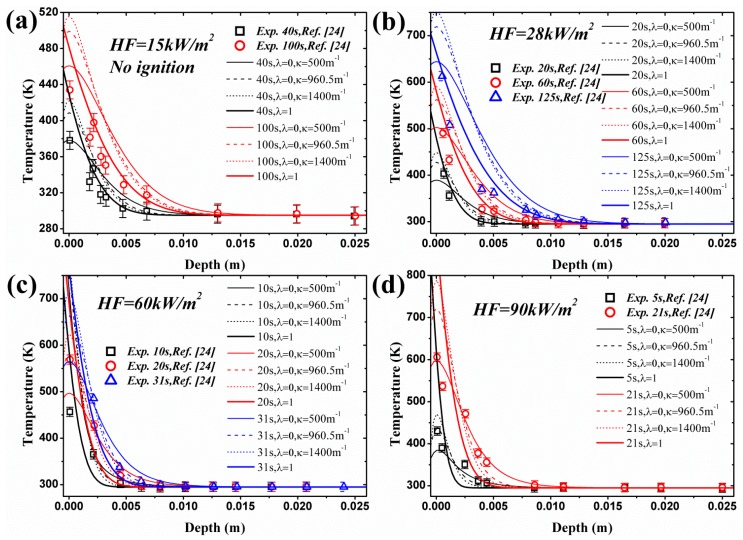
Comparison of transient temperature of coated black PMMA between surface and in-depth absorption: analytical and experimental results: (**a**) under heat flux of 15 kW/m^2^, no ignition occurs; (**b**) under heat flux of 28 kW/m^2^, ignition occurs; (**c**) under heat flux of 60 kW/m^2^, ignition occurs; (**d**) under heat flux of 90 kW/m^2^, ignition occurs.

Incident heat flux absorption mode, surface, in-depth or a combination of these determines the temperature distribution in materials. According to Equations (4), (7) and (10), transient temperature profiles can be obtained with different λ when considering in-depth absorption. Comparison between experimental and analytical models of coated black PMMA was conducted by Beaulieu [23,24], in which only surface absorption is considered and the agreement is qualitatively good. However, Jiang’s work [26] indicates this coated layer does not cancel the in-depth absorption, and this assertion has been confirmed by Bal [19]. Delichatsios added the in-depth absorption effects and developed the analytical model (e.g., [28]), but no validation is performed. In this paper, the experimental measurements of Beaulieu [23,24] are employed to compare with the analytical model, Equation (7), considering the in-depth absorption, as shown in Figure 4. The analytical results of surface absorption are also plotted in Figure 4. Three values of in-depth absorption coefficients reported in literatures are used, namely 500 m^−1^ [19] for uncoated black PMMA, 960.5 m^−1^ [26] for uncoated black PMMA and 1400 m^−1^ [19] for coated black PMMA. In Figure 4a, HF (Heat Flux) = 15 kW/m^2^, which is lower than the critical heat flux, no ignition occurs. Under all heat fluxes, the maximum value of temperature in a solid exists at the surface, depth = 0, for surface absorption. The temperature decreases sharply within the heat penetration layer estimated by $\sqrt{\alpha t}$, and then decreases slowly as the depth becomes larger. Nevertheless, for in-depth absorption the peaks of temperature profiles are present below the surface, which is also found recently by Bal [19] and Gong [29]. This phenomenon implies that ignition temperature criterion is not reasonable for in-depth absorption. After this peak, the temperature also decreases greatly in the heat penetration layer estimated by 1/κ [31]. Beyond this region, beneath the penetration layer, in-depth absorption curves surpass the surface absorption ones and the analytical model overestimates the temperature.

The agreement in Figure 4 between experimental and analytical predictions of surface absorption is better than that of in-depth absorption due to the coated layer in tests. However, the analytical model overestimates the surface temperature in Figure 4 for surface absorption, especially for higher heat flux. It also underestimates the surface temperature for in-depth absorption, especially for the lower radiation extinction coefficient, κ, like 500 and 960.5 m^−^^1^. This certainly affects the ignition time prediction of the analytical model based on the fact that both Equations (7) and (10) use the ignition temperature criterion. The predicted ignition times by Equation (14) for classical ignition theory, Equation (15) for in-depth absorption under low heat flux, Equation (16) for in-depth absorption under high heat flux and experimental results are listed in Table 2. The comparison certifies the overestimates and underestimates found in Figure 4. The predicted value for κ = 500 and 960 m^−1^ is larger than the experimental results especially under low heat flux. This is caused by the coated layer used in tests, and thus the larger radiative extinction coefficient compensates the effects of this layer.

**Figure 5:**
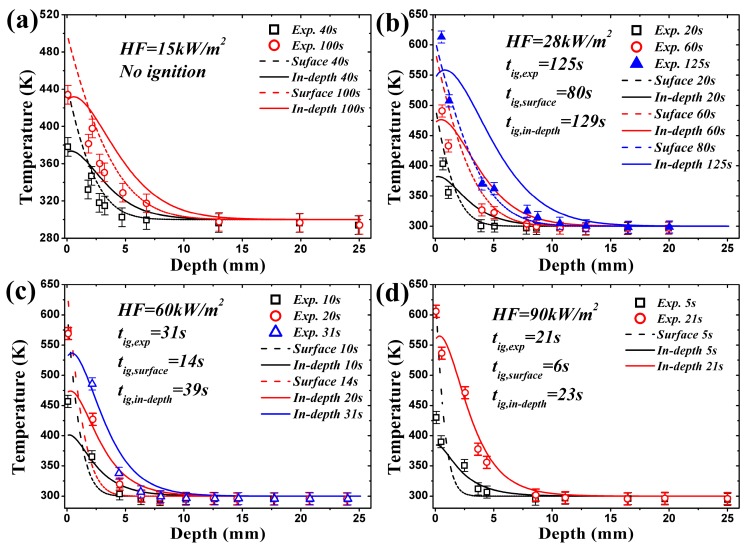
Comparison of transient temperature of coated black PMMA between surface and in-depth absorption: numerical and experimental results: (**a**) under heat flux of 15 kW/m^2^, no ignition occurs; (**b**) under heat flux of 28 kW/m^2^, ignition occurs; (**c**) under heat flux of 60 kW/m^2^, ignition occurs; (**d**) under heat flux of 90 kW/m^2^, ignition occurs.

Simulated temperature curves are illustrated in Figure 5 along with the experimental results under the same four heat fluxes. The calculated temperature, employing critical mass flux criterion, with in-depth absorption is close to the measured values in tests. However, the temperature with surface absorption is much lower compared with experimental data, which again verifies the conclusion that the in-depth absorption must be considered in investigating the ignition mechanism of translucent materials. In Figure 5b–d, only temperature distribution at ignition time with surface absorption is plotted based on the fact that the simulated *t_ig_* is lower than the measured one, such as 80 s < 125 s under 28 kW/m^2^ in Figure 5b. For all circumstances, before or at ignition time, the numerical model overestimates and underestimates the surface temperature, depth = 0, for surface and in-depth absorption, respectively. Non-monotone and monotone decreasing curves exists for in-depth and surface absorption, respectively. Beneath the heat penetration layer, the numerical model also overestimates the temperature. All these conclusions are exactly the same as the ones obtained from analytical model.

**Figure 6:**
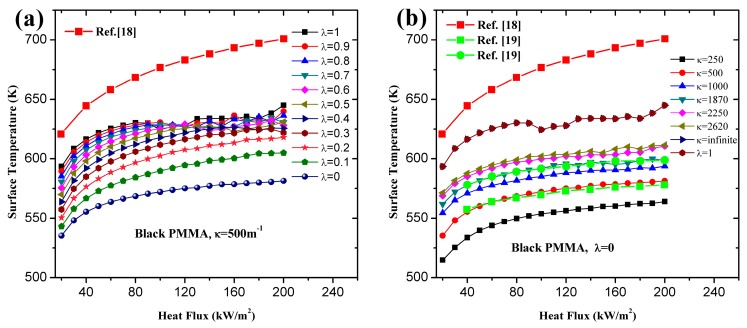
Influence of heat flux on surface temperature of black PMMA at ignition time: (**a**) different λ; (**b**) different κ.

**Figure 7:**
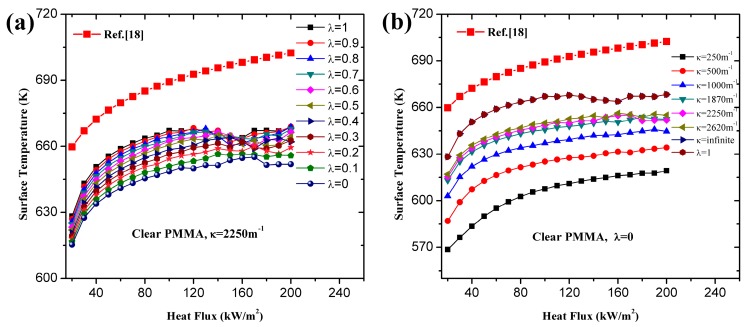
Influence of heat flux on surface temperature of clear PMMA at ignition time: (**a**) different λ; (**b**) different κ.

Surface temperature, utilized as the ignition criterion in classical theory, is reinvestigated here with the numerical method. Instead of constant ignition temperature, Lautenberger [18] proposed an approximate analytical solution for ignition of solid combustibles considering the endothermic pyrolysis taking place in materials:

(22) $$T_{ig}=T_{0}{\left(\frac{E_{s}^{\nu }{\dot{q}}_{ext}^{\prime \prime }{\dot{m}}_{cri}^{\prime \prime }}{R^{\nu }k_{s}\rho \mu A_{s}T_{0}e^{-E_{s}/RT_{1}}}\right)}^{RT_{2}/E_{s}}$$

where $\mu =341.3,\;\nu =0.85,\;T_{1}=357K,\;T_{2}=615K$ are constant values determined by the thermal parameters of black PMMA. Black PMMA is manufactured by adding a small quantity of stain agent, which means the thermal characteristics keeps almost unchanged but the optical property is affected greatly. Thus the same $\mu ,\;\nu ,\;T_{1}$ and $T_{2}$ are used in calculating the ignition time of clear PMMA. The relationships between surface temperature and heat flux of black and clear PMMA with different λ and κ are shown in Figure 6 and Figure 7. The surface temperature of PMMA at ignition time increases with incident heat flux when using critical mass flux as the ignition criterion, which agrees with the conclusions of other researchers. The increasing rate is higher for low heat flux and decreases gradually as the heat flux goes larger. Higher surface temperature caused by larger λ and κ suggests that the opacity of materials contributes to the higher temperature. Obviously, λ=0,κ=∞ gets exactly the same simulation results of λ=1. The overestimated surface temperature by Equation (22) is attributed to the negligence of heat of vaporization and convective and radiation heat loss from surface [18]. The numerical solution is in good accord with the results of ref. [19] for κ = 500 and 1400 m^−1^.

### 4.3. Optimization of λ

As discussed above, the fraction of surface absorption, λ, affects the ignition time, transient temperature and some other pyrolysis behaviors significantly before ignition. Given a fixed in-depth absorption coefficient, κ, an optimized λ can be computed by fitting the experimental data with analytical or numerical results. With this thought, Bal [19] fitted the measured $1/\sqrt{t_{ig}}$ of coated black PMMA found in the literature for κ = 500 m^−1^ with a numerical model, and an optimum value of 0.35 was obtained. Similarly, Staggs [27] obtained optimized λ = 0.52 for κ = 666.7 m^−1^ by $1/\sqrt{t_{ig}}$. Moreover, λ = 0.56 is also derived for κ = 1000 m^−1^ by experimental mass loss rate under heat flux of 50 kW/m^2^.

**Figure 8:**
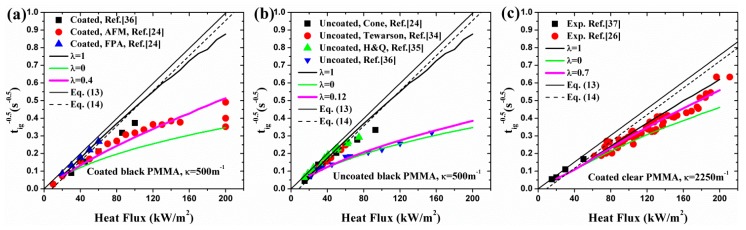
Optimized λ by experimental and numerical $1/\sqrt{t_{ig}}$: (**a**) coated black PMMA; (**b**) uncoated black PMMA; (**c**) coated clear PMMA.

In this section, the optimization of λ is performed by $1/\sqrt{t_{ig}}$, mass loss rate, and temperature distribution. Figure 8 shows the best fit of comparison between experiment and simulation results of $1/\sqrt{t_{ig}}$. κ = 500 and 2250 m^−1^ are used for black and clear PMMA (e.g., [6,19]), respectively. λ = 0.40 and 0.12 of simulation results fit the experimental measurements best for coated and uncoated black PMMA, which suggests that the coated black carbon layer enhances the surface absorptivity by 0.28, differing slightly from the value of 0.35 in ref. [19]. For coated clear PMMA, the numerical prediction with λ = 0.70 fits the experimental results best. It should be noted that the obtained optimum value of λ depends on the corresponding radiative extinction coefficient κ. Also, as suggested by Staggs [27], the validity of the model could be promoted by considering the heat flux spectrum for a fixed λ.

**Figure 9:**
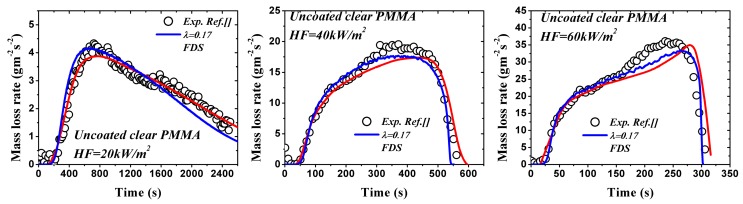
Optimized λ by experimental and numerical mass loss rate of uncoated clear PMMA under different heat fluxes.

Measured mass loss rate in tests can also be employed to optimize λ. Since the optimization process has been conducted in ref. [27] for coated black PMMA, only the comparison between the numerical model and experimental data of uncoated clear PMMA available in ref. [29] is exhibited in Figure 9. The optimized λ = 0.17 is obtained according to the residual analysis for κ = 2250 m^−1^. The agreement between experimental, numerical and FDS [9] is fairly good. Combining with the corresponding optimized λ = 0.70 for coated clear PMMA discussed above, it can be concluded that the coated layer increases the surface absorptivity by 0.53 for clear PMMA. Obviously, this value is much larger than that of black PMMA, which may be mainly caused by the difference between in-depth absorption coefficients of materials, spectral characteristics of heater, optical properties and thickness of the painted black layer used by each researcher.

The measured transient temperature plotted in Figure 4 and Figure 5 can also be used to obtain the optimized λ by fitting the analytical or numerical results. Table 3 lists the corresponding values of optimized λ under different incident heat flux of coated black PMMA for κ = 500 m^−1^. λ decreases with increasing heat flux for both analytical and numerical results, which indicates that the surface and in-depth absorption are the dominant mechanisms for low and high heat flux, respectively. The discrepancy between analytical and numerical optimized λ is attributed to the constant and temperature-dependent thermal parameters which are important in determining the temperature profiles.

## 5. Conclusions

The combination of surface and in-depth absorption of incident heat flux imposed on PMMA has a significant influence on ignition behaviors. Theoretical analysis and a numerical model developed by authors previously are employed to study the effects. Based on the comparison with experimental measurement, including black and clear PMMA, some important conclusions are obtained and summarized as follow:

1. Utilization of constant or variable thermal parameters has little effect on ignition time for a wide range of heat fluxes, which agrees with the conclusion of Bal [19]. For both black and clear PMMA, linearity of $t_{ig}^{-0.5}$ increases with increasing surface absorptivity, λ. In-depth absorption contributes to the curve, especially under high heat flux. For pure in-depth absorption, the analytical model results fit the numerical ones well with different radiative extinction coefficient values.
2. For surface absorption, surface temperature is the maximum value in a solid, while for in-depth absorption, the peak exists below the surface and the surface temperature cannot be used as the ignition criterion. Both analytical and numerical models overestimate the temperature in the heat penetration layer and underestimate the temperature beyond this region for surface absorption. The opposite is true for in-depth absorption. Affected by this overestimation, the predicted ignition time is much lower than the measured value in tests due to the fact that the critical temperature is used as the ignition criterion in the analytical model. However, the agreement between numerical and experimental results is much better when the critical mass flux is used. The surface temperature increases with increasing heat flux, and the increasing rate declines as the heat flux gets larger. Furthermore, larger λ and κ, contributing to the opacity of material, also lead to higher surface temperature.
3. The best combination of these two absorption modes is explored for black and clear PMMA, including coated and uncoated conditions, by ignition time, mass loss rate and transient temperature in material. The optimized λ was found to be 0.40 and 0.12 for coated and uncoated black PMMA, leading to an enhancement of surface absorptivity by 0.28. While for clear PMMA, the optimized λ was computed to be 0.70 and 0.17 for coated and uncoated ones, respectively, resulting in an increase of 0.53 in the painted black layer. Moreover, the optimized λ decreases with increasing incident heat flux based on the analytical and numerical calculations.

## Figures and Tables

**Table 1:** Parameters of PMMA used in the simulation and analytical model.

Parameters	Black PMMA		Clear PMMA	
	Values	Ref.	Values	Ref.
Density, *ρ*(kg/m^3^)	1187.8	[19]	1200	[27]
	$1404.5-0.7316T_{s}$	[30]	$\partial \rho_{s}/\partial t=\rho_{s}S_{v}$	[29]
Pre-exponential factor, *A_s_* (1/s)	5 × 10^8^	[19]	8.6 × 10^12^	[6]
Activation energy, *E_s_* (J/mol)	1.25 × 10^5^	[19]	1.88 × 10^5^	[6]
Heat of vaporization, *∆H_v_* (J/g)	900	[23]	846	[6]
Absorption coefficient, *κ* (1/m)	500	[19]	1000	[27]
	960	[26]	1870	[31]
	1400	[19]	2250	[6]
Specific heat, *C_s_* (J/(g·K))	1.665	[19]	1.7	[27]
	$\left\{ \begin{array}{ll} 0.173+204.1T_{s}^{-2}+0.0043T_{s}, & T_{s}<378\\ 1.212+0.00255T_{s}, & T_{s}\geq 378 \end{array}\right.$	[30]	0.6 + 0.00367*T_s_*	[6]
Thermal conductivity, *k* (J/(s·m·K)	0.21	[19]	0.2	[27]
	$\left\{ \begin{array}{l} 0.1824+4.954\times 10^{-5}T_{s},T_{s}<378\\ 0.2882-2.318\times 10^{-4}T_{s},T_{s}\geq 378 \end{array}\right.$	[30]	$\left\{ \begin{array}{l} 0.45-0.00038T_{s},T_{s}<378\\ 0.27-0.00024T_{s},T_{s}\geq 378 \end{array}\right.$	[6]
Reflectivity, *r* (-)	0	[19]	0.05	[6]
Critical mass flux at ignition, ${\dot{m}}_{ig}^{\prime \prime }$ (g/m^2^·s)	2.42	[19]	2.5	[9]
			4.5	[32]
Convection coefficient, *h* (J/sm^2^·K)	10	[19]	5	[6]
	Equation(15)	[29]	25	[27]
			Equation(15)	[29]
Ambient temperature, *T_∞_*, (K)	300	*	300	*

* Experimentally measured value.

**Table 2:** Comparison of ignition time between experimential and analytical models.

${\dot{q}}_{ext}^{\prime \prime }$(kW/m^2^)	*t_ig_(s)*				
	Experiential data Ref. [23]	Equation (15), λ = 0 *κ =* 500 m^−1^	Equation (15), λ = 0 *κ =* 960.5 m^−1^	Equation (15), λ = 0 *κ =* 1400 m^−1^	Equation (14), λ = 1
28	125	247.35	172.16	149.59	105.84
60	31	87.17	45.28	34.10	15.19
90	21	62.45	28.03	19.41	6.07
${\dot{q}}_{ext}^{\prime \prime }$kW/m^2^)	Experiential data Ref. [23]	Equation (16), λ = 0 *κ =* 500 m^−1^	Equation (16), λ = 0 *κ =* 960.5 m^−1^	Equation (16), λ = 0 *κ =* 1400 m^−1^	Equation (14), λ = 1
28	125	202.36	158.83	143.08	105.84
60	31	48.28	33.64	28.31	15.19
90	21	25.94	17.18	14	6.07

**Table 3:** Optimized λ by measured transient temperature.

HF (kW/m^2^)	15	28	60	90	Average
Analytical	0.77	0.63	0.47	0.23	0.53
Numerical	0.54	0.49	0.38	0.21	0.41
